# Vibroacoustical Performance Analysis of a Rigid Device Casing with Piezoelectric Shunt Damping

**DOI:** 10.3390/s21072517

**Published:** 2021-04-04

**Authors:** Krzysztof Mazur, Jaroslaw Rzepecki, Anna Pietruszewska, Stanislaw Wrona, Marek Pawelczyk

**Affiliations:** Silesian University of Technology, Department of Measurements and Control Systems, Akademicka 16, 44-100 Gliwice, Poland; Krzysztof.Jan.Mazur@polsl.pl (K.M.); Anna.Pietruszewska@polsl.pl (A.P.); Stanislaw.Wrona@polsl.pl (S.W.); Marek.Pawelczyk@polsl.pl (M.P.)

**Keywords:** piezoelectric shunt damping, rigid device casing, synchronized switch damping, vibration damping, sound pressure level reduction

## Abstract

Noise and vibration are common issues that may have a negative impact on human’s’ health. To minimize their consequences, several vibroacoustical methods may be employed. One well-known method is Piezoelectric Shunt Damping (PSD). Over the years, many approaches have been investigated, from passive, state switching circuits to active pulse-switching. In this paper, the authors propose three PSD implementations—passive Synchronized Switch Damping on Inductor (SSDI), semi-active SSDI and active Synchronized Switch Damping on Voltage source (SSDV)—for a single-panel structure mounted on a rigid-frame casing. The nine Macro Fiber Composite (MFC) elements were mounted on the plate based on preliminary simulations in FreeFEM. Then, the theoretical results were validated by an identification experiment. The main research is concentrated on the Sound Pressure Level (SPL) and structural vibrations reduction for selected frequencies. The active method provided the highest reduction of vibration—up to 5.5 dB for maximal possible loudspeaker level without overdrive and up to 7.5 dB for lower excitation levels.

## 1. Introduction

Noise and vibration constitute a serious issue in the human environment [1]. Duration of exposure to noise, as well as its frequency, are crucial due to their impact on human health [2]. Possible consequences of prolonged exposure to environmental noise may include, among others, lower mental performance [3], stroke, heart disease, depression and lower job performance [4,5]. Noise is frequently a result of structures’ vibration. Vibration is common in domestic appliances, transportation, and manufacturing [6]. Exposure to vibration may be especially harmful for human health at low frequencies, up to 100 Hz [7].

There are three widely recognized main types of methods of noise and vibration control: active, semi-active and passive.

In the passive approach, noise may be reduced with the use of, e.g., mufflers, barriers, silencers [8], panels with rib reinforcements [9], and structure-borne noise, or vibration may be reduced either with the use of isolators or damping materials, Helmholtz resonators [10], or by changing system’s structure [8]. A disadvantage of passive methods is an increase of dimensions of the barriers and their weight [11]. Their effectiveness drops when the frequency decreases. In such situations, semi-active or active methods may be alternative approaches to reduce noise and/or vibration.

Active methods are characterized by high efficiency at low frequencies. They may be implemented in the control systems complementary to different solutions or as an alternative [12,13]. A well-known approach is Active Noise Control (ANC), which has been rapidly developing since 1990s. In this approach, loudspeakers are usually secondary sources. At low frequencies, this approach may generate a lower economic burden in comparison to passive methods [12]. However, ANC may result in achieving local zones of quiet only, while noise reinforcement is observed elsewhere. An alternative to ANC is Active Noise-Vibration Control (ANVC), where flexible walls are excited by strain or inertial actuators to obtain a desired acoustic control source. Another approach, where structural actuators (for instance, shakers or piezoelectric elements [14,15]) are integrated into the walls, is Active Structural Acoustic Control (ASAC) [16], which achieves sound reduction in a different way than ANVC. An important step in the development of this approach was to integrate error sensors within the enclosure boundaries.

Besides active and passive methods, the semi-active (also known as semi-passive) approach may also be employed to combine their benefits  [17]. Semi-active methods can adapt to the changes of environmental conditions [18].

One of the most successful approaches to reduce noise and vibration can be a casing enclosing a noise-generating device. In their previous research, the authors employed mainly two different types of the casings: the elastic (lightweight) one  [19], built of thin plates bolted together, and the rigid one [20], which may be built of single or double panels mounted on a rigid frame. The active control with the use of active casing was previously applied by the authors, and its efficiency was reported in many scientific articles, e.g., [20]. The authors applied active control also to real devices [21]. A theoretical study of a double-panel rigid device casing was also provided, and mathematical models were developed as a result [22].

Another proposal for classification of methods relates to the reduction of structural vibration with the use of piezoelectric materials, which have excellent electromechanical coupling characteristics [23]. Two main groups are distinguished in this case, i.e., Piezoelectric Shunt Damping (PSD) and classical Active Vibration Control (AVC) [24]. In some of the active vibration reduction systems, high energy consumption is observed, e.g., in active suspension systems [25].

PSD can be further divided into active, semi-active and passive methods. In PSD, piezoelectric materials are driven by an electric circuit [26]. Passive approaches, such as linear resonant shunt, have serious drawbacks, e.g., sensitivity to environmental variations and large inductance requirements for the low-frequency applications [27]. An alternative hybrid solution may be SSSA (synchronized switched shunt architecture) [28]. A widely used semi-active approach to PSD is SSD (Synchronized Switch Damping) [27]. Several variations of SSD methods exist, e.g., SSDS (SSD on short circuit), SSDI (SSD on Inductor), SSDV (SSD on voltage source) or SSDNC (SSD on negative capacitance) [29]. However, the advantage of active techniques is their robustness and high control performance [30].

PSD methods are based on piezoelectric materials’ property of producing electrical energy under the straining. The elements are able to absorb mechanical energy and to convert it into the electric charge, stored in internal capacity. The main challenge is to dissipate this energy efficiently. PSD can be also divided, based on techniques of energy dissipation. In the literature, two main approaches can be found.

The first approach is based on passive circuits, which means that the whole system cannot be supplied by external energy. The easiest way is adding a resistance, which loses energy by itself as heat emission. Another method is to build a simple, shunt-resonant circuit, based on the internal capacitance of piezoelectric element, additional resistance and inductance [31]. There are many variations of such circuits: from serial RL, through the parallel version,  to additional capacitance, proposed by Fleming et al. [32]. The main advantage compared to the previous solutions is a significant decrease of required inductance in the circuit. In practice, higher values of inductance can be implemented only with the use of the high-voltage op-amps. Such a solution requires external energy source, which means that this method is active [33].

The second approach is based on switching circuits. One of the methods is a state-switching between the open circuit, when the piezoelectric elements absorb mechanical energy, and the short circuit, for dissipation of the electric charge  [34]. An alternative method is pulse-switching, where generated charge is added to the piezoelectric element, after inversion on the RL circuit. In this case, the piezoelectric element acts similarly to an actuator in the active system  [35]. A similar technique has been proposed by Richard et al. [36]. In the literature, it is widely recognized as SSDI. The efficiency of this method is significantly correlated with electromechanical coupling of the vibrating structure. In 2006 Lefeuvre et al. proposed a new method to overcome this issue called SSDV [37]. The improvement of damping is based on artificially increasing the voltage, inversed on the resonant circuit with the use of additional voltage sources. The use of external energy makes it an active method. The switching circuits are more beneficial, such as Hagood’s solution, due to its low inductance value. Efficiency of such solutions depends mainly on voltage amplitude on the piezoelectric element [38].

In recent years, many different approaches to shunt systems have been studied, and novel methods have been introduced. For instance, Zhang et al.  [39] employed the SSD technique with an enhanced analog circuit to produce significant damping for multiple modes. Pohl [40] proposed an improved negative capacitance shunt damping system with optimized characteristics for use with piezoelectric transducers. Pohl and Rose [41] examined a new damping concept for circular saw blades, achieving good reduction of vibration amplitude over a wide frequency range in the non-rotating condition. Silva et al. [42] studied the SSDI damping structure and used it as a wideband energy harvester. Wang et al. [43] proposed a fuzzy adaptive SSDV method to effectively shorten the vibration damping time and reduce the displacement. Wu et al. [44] developed a linearization method for SSD to increase the computational efficiency. Asanuma and Komatsuzaki [45] proposed a new method to predict the attenuation performance in a partially covered piezoelectric cantilever connected to a self-sensing SSDI circuit. Lallart and Lombardi [46] designed a Synchronized Switch Harvesting on ElectroMagnetic system, based on the previously developed Synchronized Switch Harvesting on Inductor (SSHI) scheme. Mohammadi et al. [47] introduced a novel, powerful approach to SSD on Capacitor (SSDC) vibration suppression with the use of magnetostrictive materials instead of piezoelectrics. Wu et al. [48] proposed a new type of SSD—SSD with Diode (SSDD) for enhanced vibration damping of smart structure, with diodes and switches used to form a combinational network. Ji et al. [49] developed new SSD control method—an unsymmetrical SSD. They also invented and examined a method of realizing unsymmetrical bipolar voltage with an SSD method based on negative capacitance shunt circuit, for  improvement of the vibration control performance [50]. Zhang et al. [51] proposed an enhanced SSDI (synchronized switch damping on inductor) approach to suppress the vibration of bladed disks in aero-engines.

The authors employed a passive aproach in the rigid casing with the use of a shunt system, based on SSD on Inductor (SSDI) control law [18]. Recently, the authors examined the rigid casing coupled with the use of electromagnetic elements, where spectral analysis of vibration was conducted based on Macro Fiber Composites (MFC) measurements [52].

In this paper, the authors present results of comparison of passive SSDI, semi-active SSDI and active SSDV. The passive SSDI implementation is based on previous implementations proposed by the authors  [18,53]. Based on previous simulations  [53], the circuit has been improved. In this paper, the first experimental results of the improved circuit are reported. Additionally, beside the vibration reduction, the authors considered also influence of PSD on SPL reduction. It is shown that the proposed passive SSDI circuit also reduces the SPL outside the casing. To improve SSDI performance further, a novel microprocessor-based semi-active SSDI implementation has been proposed. In this implementation, the small energy needed to drive Metal-Oxide Semiconductor Field Effect Transistor (MOSFET) switches comes from external power source. This switch provides a smaller voltage drop and improves switching efficiency. Additionally, digital implementation of SSDI switching law introduces smaller delay and is more robust. The third circuit, the active SSDV circuit, is a minimal modification of the semi-active SSDI circuit that significantly improves performance. Compared to classical SSDV implementation, the voltage boost in provided only on one voltage transition, from negative to positive voltage on MFC. This novel approach provides performance improvement at no cost compared to the semi-active approach. Full SSDV implementation would require an additional power supply rail.

The paper is organized as follows: Section 2 describes the rigid device casing with a single-panel structure, measurement system and vibration control system. In Section 3, experiments are described, and results are presented. Section 4 provides discussion of the obtained effects. Section 5 is a summary of the paper, containing conclusions drawn from the performed experiments.

## 2. Materials and Methods

### 2.1. Experimental Setup

**Figure 1:**
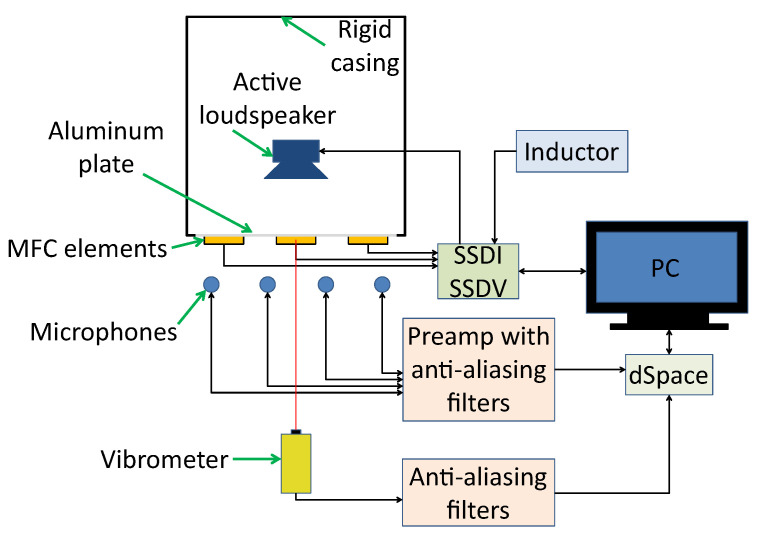
A scheme of the experimental setup.

**Figure 2:**
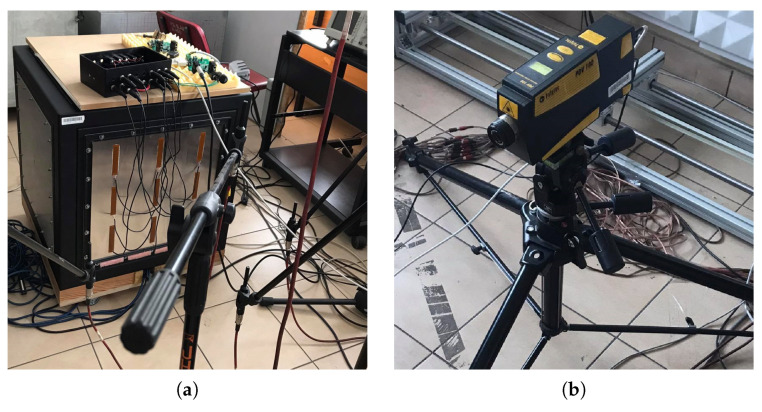
Pictures of the equipment used in the experiments: casing with MFC elements (**a**), laser Doppler Vibrometer (**b**).

The main part of the experimental setup (Figure 1) is a cubic rigid casing with 0.6 m × 0.6 m × 0.6 m dimensions. The MFC elements are attached to a plate, which is the front wall investigated in this paper (Figure 2a). The plate was excited to vibrate using active loudspeaker emitting a tonal signal, provided by generator, integrated with the SSDI plate.

**Figure 3:**
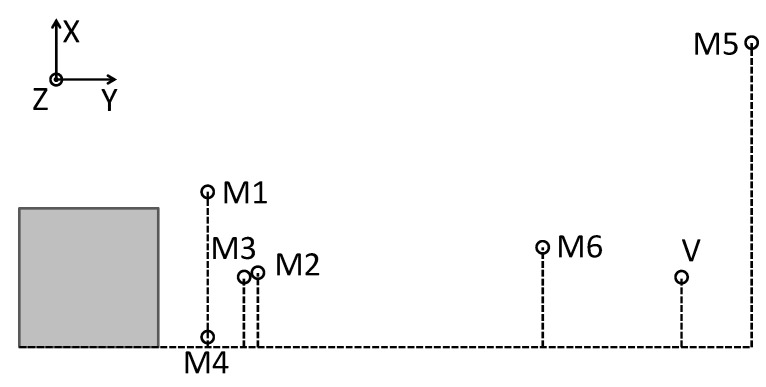
Top view scheme of placement of the casing, microphones and vibrometer.

The vibroacoustical signal was measured using a laser Doppler vibrometer (Figure 2b), focused on the central point of vibrating panel and six microphones, distributed arbitrarily (Figure 3). M1–M4 measure sound emission from vibrating plate, and M5, M6 are used to measure SPL in distant locations in the laboratory. The origin of the coordinate system is on the lower-left corner in the rear wall of the casing. The (x,y,z) coordinates are presented in Table 1. Both types of sensors are connected to dSpace DS1104 Controller Board, through the anti-aliasing filters with 3 dB corner frequency at 480 Hz. The signals are acquired with 16 kHz sampling frequency, digitally resampled to 2 kHz. The experiments were performed in a laboratory equipped with acoustic diffusers and absorbers, because microphone measurement is sensitive to reflections.

The setup was controlled remotely and allowed to create autonomous measurement system, working accordingly to preliminarly defined scenarios.

### 2.2. Rigid Casing with a Single-Panel Structure

In this research, a single-panel cubic casing with a rigid frame is investigated. The resonant frequencies of the front wall vibration and modeshapes were obtained using theoretical model in FreeFEM. Then, the results were validated by performing an identification experiment.

The Kirchhoff–Love plate theory is used to model the individual panels. The transverse displacement η of the panel is modeled as [54]:

(1) $$\begin{array}{c} D\left(\frac{\partial^{4}\eta \left(x,y,t\right)}{\partial x^{4}}+2\frac{\partial^{4}\eta \left(x,y,t\right)}{\partial x^{2}\partial y^{2}}+\frac{\partial^{4}\eta \left(x,y,t\right)}{\partial y^{4}}\right)+\rho h\frac{\partial^{2}\eta \left(x,y,t\right)}{\partial t^{2}}\\ =f_{ext}\left(x,y,t\right)+f_{mfc}\left(x,y,t\right); \end{array}$$

where $D=\frac{Eh^{3}}{12(1-\nu^{2})}$ is a panel flexural rigidly, *x*, *y* are the coordinates, *t* is a time, *h* is a thickness of panel, ρ is a density of panel’s material, $f_{ext}$ is a lateral external loading and $f_{mfc}$ is a lateral MFC loading.

The examined part is a 1.0 mm aluminum plate, mounted as a front wall on a rigid, heavy frame, employed in previous research conducted by the authors  [18,20,52]. The plate is clamped using twenty screws and by an additional square frame, to provide boundary conditions close to fully clamped. The model used in simulation uses ideal fully clamped boundary conditions:

(2) $$\forall x,y\in \partial \Omega \;\eta \left(x,y,t\right)=0,\frac{\partial \eta (x,y,t)}{\partial x}=0,\frac{\partial \eta (x,y,t)}{\partial y}=0,$$

where ∂Ω is the boundary of a plate.

The other (top and side) walls are made of thicker (3.0 mm) aluminum panels to enhance the casing’s acoustic insulation. In this research, the performance analysis was conducted only for the front wall with the assumption that the interseting frequencies are propagating mainly from that direction.

**Figure 4:**
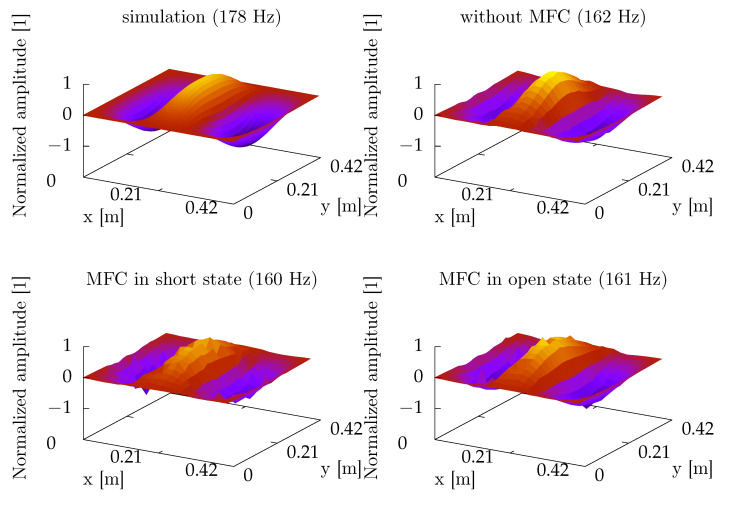
A comparison of (3,1) modeshape obtained from FreeFEM simulation and real experiments.

**Figure 5:**
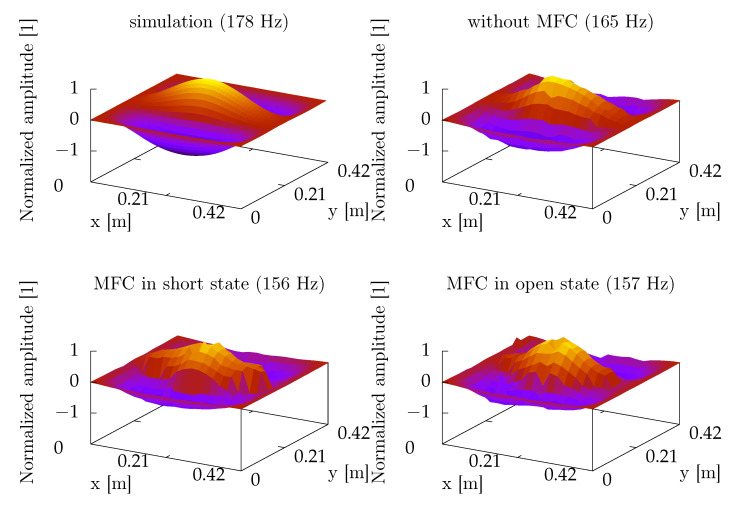
A comparison of (1,3) modeshape obtained from FreeFEM simulation and real experiments.

During the identification experiment, the plate was excited by a wideband noise up to 5.6 kHz. The vibration was measured over a grid of 12 × 12 points, regularly distributed on the plate, by an automatic positioning system carriage with a laser vibrometer [55]. The results were used to determine frequencies of modes, which were important for the further experiments. The highest amplitudes were obtained for (3,1) (Figure 4) and (1,3) modes (Figure 5). The differences between modal frequencies predicted by the model and measured in experiment are quite significant. The model predicts the same frequency for (3,1) and (1,3) modes, but in experiments, different frequencies were obtained, consecutively 7% and 9% lower than the frequency predicted by the model. The model can be tuned to both frequencies obtained in the experiment by assuming non-ideal boundary conditions.

Nine MFC elements were attached at every anti-node of the (3,3) mode using epoxy glue. This allowed us to test both the (1,3) and (3,1) modes. Based on previous research [53], the authors decided to connect the elements in series to sum the voltage amplitudes, and polarization of the MFCs, attached in the second line, was inverted to obtain the same phase of output signal of every element. This solution increased the value of voltage obtained from MFCs. The main parameters of the aluminum plate and MFC elements are presented in Table 2. MFC elements used in the experiments are of type M8514-P2, manufactured by Smart Material company. They are characterized by different values of Young’s modulus in both the rod and the electrode direction. This is expressed in Table 2 as a/b, where *a* is Young’s modulus in the the rod direction, and *b* is Young’s modulus in the electrode direction.

**Figure 6:**
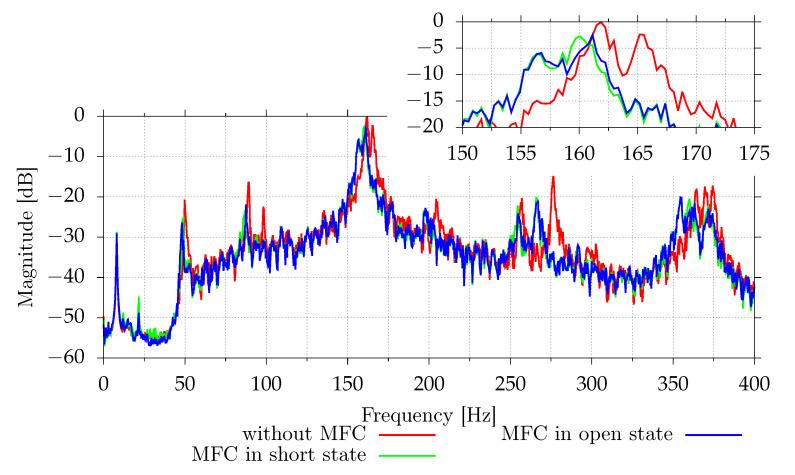
Comparison of mean vibrations of the 1.0 mm aluminum plate without MFCs and with MFCs.

Vibrations of the investigated plate were measured before and after MFC mounting (Figure 6). The results are similar, without any noticeable shifts of resonant frequencies between MFC in the open and short states. However, differences between frequency characteristics before and after MFC mounting are significant. Besides the frequency values, it was noticed that the positions of (3,1) and (1,3) resonances are swapped. This may not be the only such situation, but this case was confirmed based on the modeshapes obtained during the identification experiment (Figure 5).

### 2.3. Vibration Damping Methods

The single-panel structure with MFC elements investigated in this paper can be represented by an equation for the 1-DOF system [18]:

(3) $$\left(M+M_{mfc}\right)\ddot{u}+C\dot{u}+\left(K_{s}+K_{mfc}\right)u=F-\alpha V_{mfc};$$

where *M* is the mass of a structure, $M_{mfc}$ is the mass of MFC element, *C* is damping factor, $K_{s}$ is structure’s stiffness, $K_{mfc}$ is MFC elements stiffness, *u* is structure’s displacement, *F* is the external force, α is a force factor and $V_{mfc}$ is the voltage on MFC elements.

**Figure 7:**
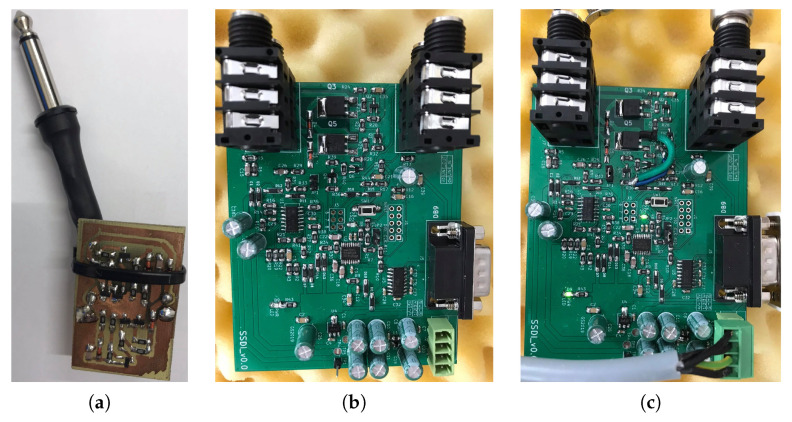
The circuits used in the experiments: passive (**a**), SSDI (**b**), SSDV (**c**).

During the experiments, three types of vibration damping circuits were investigated: passive SSDI, semi-passive SSDI and active SSDV (Figure 7). Both SSDI and SSDV are pulse-switching techniques. In the open state, the charge from the resonant circuit is applied to MFC elements, which also absorb the mechanical energy. In the short state, the transformed energy is inversed on an inductor. The main challenge in such techniques is to switch the circuit between the open and short state in an appropriate time to reach the highest efficiency. This requires applying the swiching law, based, e.g., on structure’s velocity, in which the detection of the sign changes, or on detection of maximal voltage on MFC elements.

### 2.4. Passive Shunt Circuit

**Figure 8:**
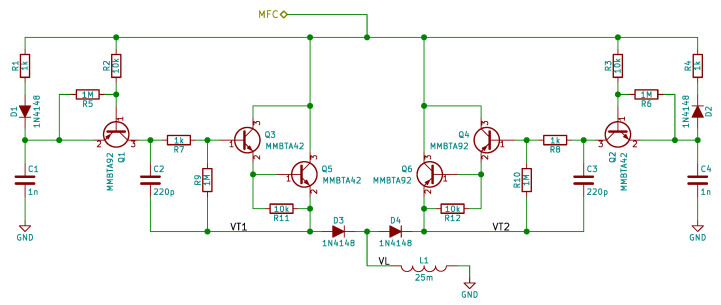
The passive SSDI electrical circuit scheme.

Figure 8 contains electrical circuit scheme for passive SSDI implementation. This implementation has been proposed earlier by the authors [53]. An earlier version of this circuit, without C2 and C3 capacitors, was also used in [18].

The circuit is connected only to MFC elements; they are connected between the “MFC” label and the ground. The circuit is composed by two complementary subcircuits, one responsible for positive-to-negative transition and the second responsible for negative to positive transition. In the first subcircuit, R1, D1 and C1 are responsible for detection of maximal voltage on MFCs. The C1 capacitor holds the maximal voltage and provides power needed by the BJT-based  switch to operate. The Q1 transistor is used to detect drop in MFC voltage caused by discharging due to change of plate velocity. R2 resistor is used to reduce base current. R5 resistor is used to reduce base leakage current when Q1 transistor should be turned off. Otherwise, the small base current could be amplified. To allow for a small C1 capacitor value, the Darlington pair (Q3, Q5 and R11) is used as a switch. R7 and R9 resistors are used to reduce the Q3 transistor base current and to avoid amplification of leakage currents, respectively. C2 capacitor is used to provide some noise immunity. Without it, at high vibration levels, the circuit occasionally switches due to oscillations caused by switching, and the vibration reduction performance is reduced. The D3 diode is used to provide protection against reverse biasing of base-emitter junctions of Q3 and Q5 transistors. In the second subcircuit, diodes are reversed and BJTs are changed to their complementary counterparts.

### 2.5. Semi-Active and Active Shunt Circuits

**Figure 9:**
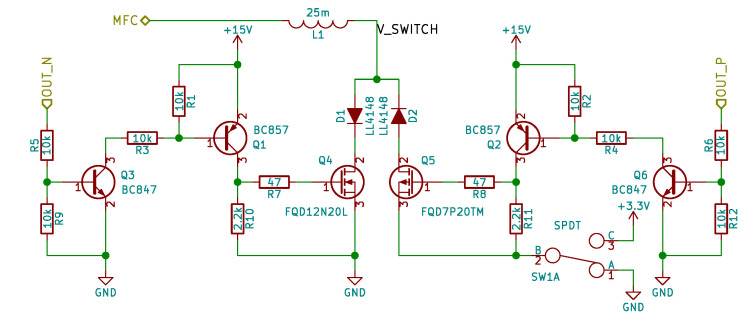
Semi-active/active shunt circuit electrical circuit scheme, MOSFET switches.

Semi-active and active SSDI/SSDV systems share the implementation, the only difference is the Q5 Metal-Oxide Semiconductor Field Effect Transistor (MOSFET) source voltage (Figure 9). In the semi-active implementation the source and gate-to-source resistor R11 is connected to ground. In active system the source is connected to 3.3 V power supply rail. This power supply rail is already available on the board and it is used by the microcontroller.

The OUT_N and OUT_P signals are generated by the microcontroller. BJTs are used to convert 3.3 V signal from microcontroller to +15 V signal or −15 V used to drive MOSFET gates. Small gate resistors are used to damp potential oscillations. D1 and D2 diodes are used to block reverse currents.

**Figure 10:**
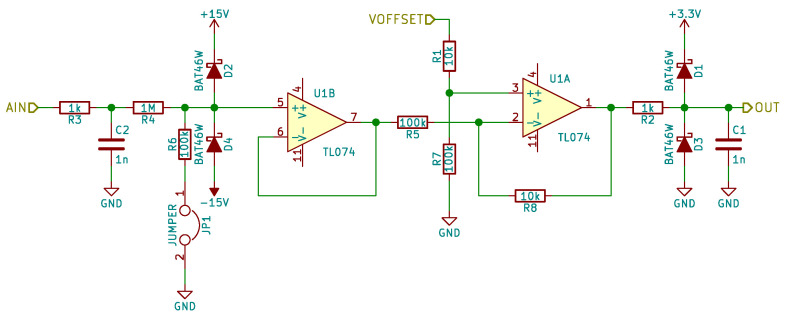
MFC voltage measurement circuit.

The voltage on switch (V_SWITCH label) is measured by the microprocessor. Figure 10 shows the circuit used to measure this voltage. R1 and C1 are used both as RFI  filters, needed to avoid RF rectification in operational amplifiers, and as a part of an anti-aliasing filter. R4 and R6 form an optional 1/11 voltage divider that extends the measurement range to ±110 V. If JP1 jumper is open, the measurement range is equal to ±10 V. D2 and D4 diodes are used to protect amplifier against too high or too low voltages. The U1B amplifier is used as a buffer. The U1A amplifier with R1, R5, R7 and R8 resistor implements a differential amplifier with 1/10 gain and adds an offset (VOFFSET equal to 1.65 V) needed to convert bipolar input signal to the unipolar signal needed by (Analog to Digital Converter (ADC). Therefore, the nominal ±10 V input voltage range is converted to 0.65 V to 2.65 V range. R2 and C1 implement a second pole of anti-aliasing filter. D1 and D2 diodes are used to protect the ADC against potential overvoltage or undervoltage. The output signal OUT is sampled by unipolar 3.3 V 12-bit ADC. The ADC operates at 1 Msps. The signal from ADC is later resampled to 100 kHz, and the SSDI control algorithm operates at 100 kHz.

## 3. Results

### 3.1. Performance for Frequencies near Resonance

**Figure 11:**
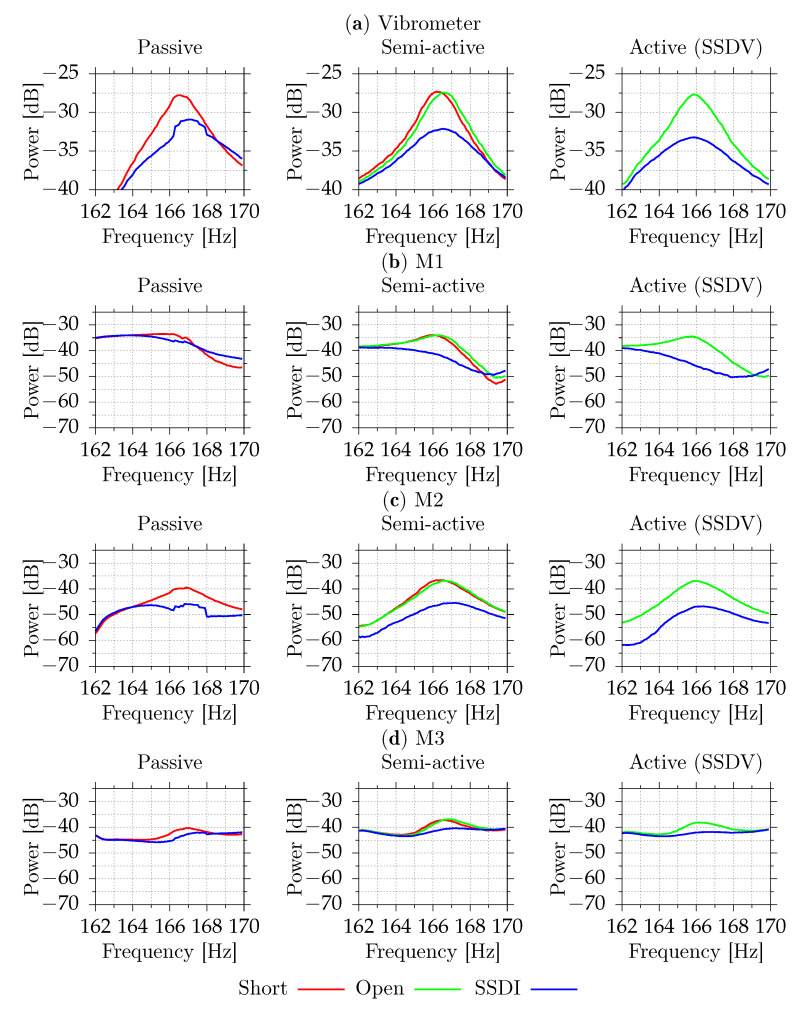
Measured vibration (**a**) and acoustic signal powers for M1 (**b**), M2 (**c**), M3 (**d**) for different excitation frequencies (part 1).

**Figure 12:**
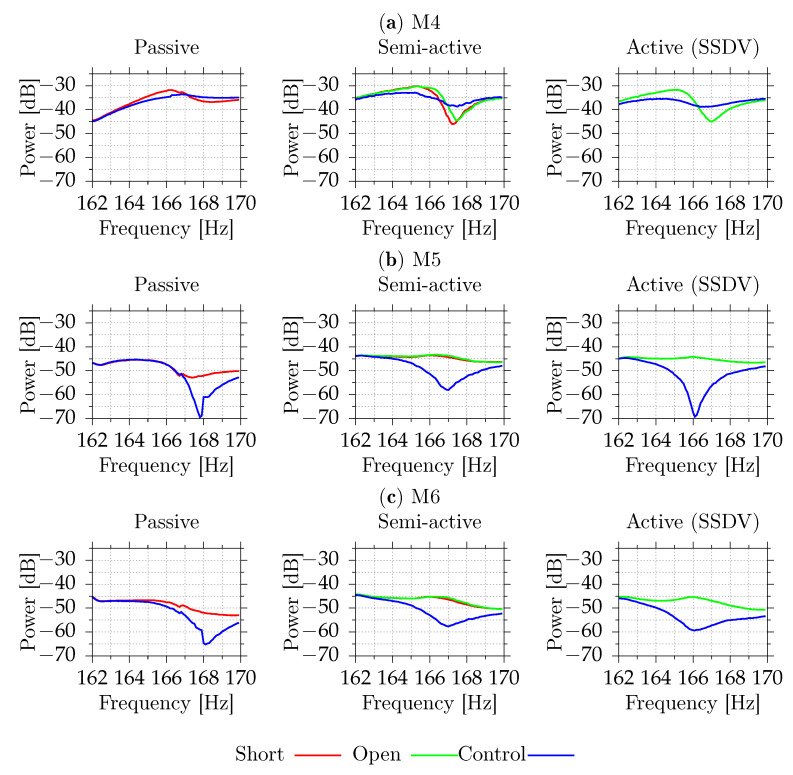
Measured acoustic signal powers for M4 (**a**), M5 (**b**), M6 (**c**) for different excitation frequencies (part 2).

Figure 11 and Figure 12 show the digital signal power measured by the vibrometer and all microphones for different excitation frequencies. Each shunt system was tested separately. Because the SSDI algorithm assumes that the excitation is tonal, systems were tested for pure tonal excitations. This algorithm may also work for other excitations as long the resulting plate vibrations are close to the tonal signal. This is true if only one mode with low damping factor is excited. For each frequency, from 162 Hz to 170 Hz, with 0.1 Hz step, the signal powers were measured with disabled and enabled shunt system. In the case of the semi-active system, two cases for disabled system were tested—open circuit and simulated short circuit (both MOSFET transistors were enabled). Each power sample was measured using 32,768 samples at 2 kHz (16.384 s). After each change, 3 s delay was added. Due to possible changes of environmental conditions, which could affect resonance frequency slightly, the result for each shunt system is presented separately.

For the tested casing, the differences between the open circuit and the simulated short circuits are small. The passive shunt system provides up to 4.8 dB reduction of the velocity level (69% reduction of power) at the point of measurement. Semi-active system provides reduction up to 5.0 dB (68%) compared to the short circuit and up to 4.7 dB (66%) compared to the open circuit. The active system increases reduction to 5.5 dB (72%) compared to the open circuit. Table 3 shows the numerical values at the open circuit resonant frequency. For the semi-active and active circuits, the best reduction is obtained close to the open circuit resonant frequency. For the passive circuit, the short circuit resonant frequency occurs at frequencies for which the vibration reduction performance is slightly degraded (Figure 11). The maximal vibration reduction, equal to 4.8 dB, is obtained at 166.2 Hz.

### 3.2. Switching Efficiency

**Figure 13:**
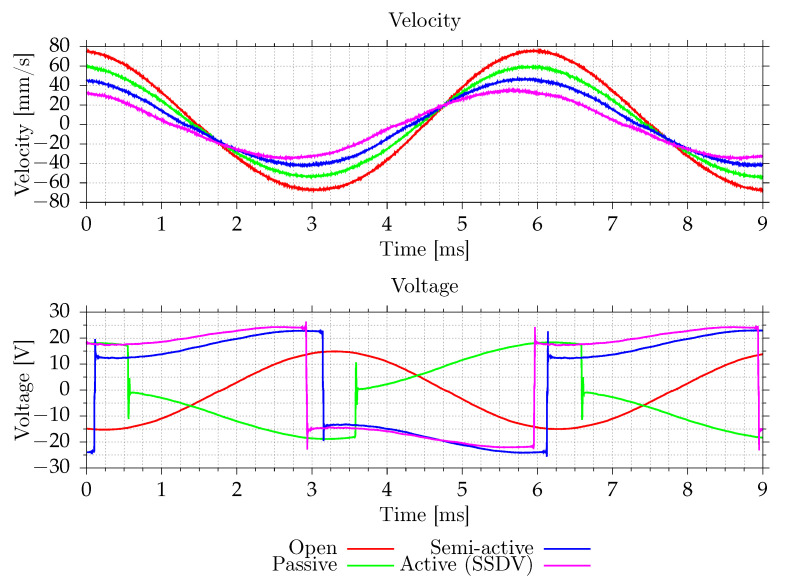
Measured plate velocity and a voltage on MFC for open-circuit resonant frequency.

**Figure 14:**
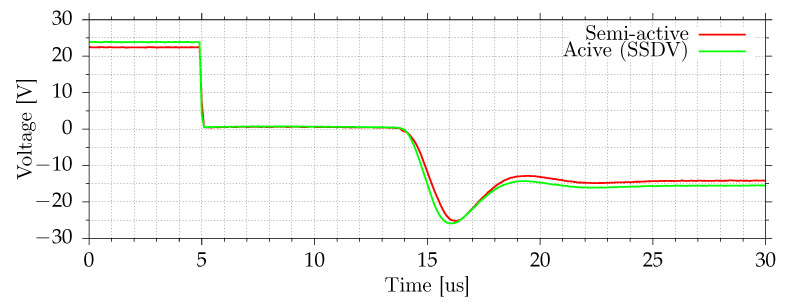
Voltage on a switch during positive to negative voltage transition.

**Figure 15:**
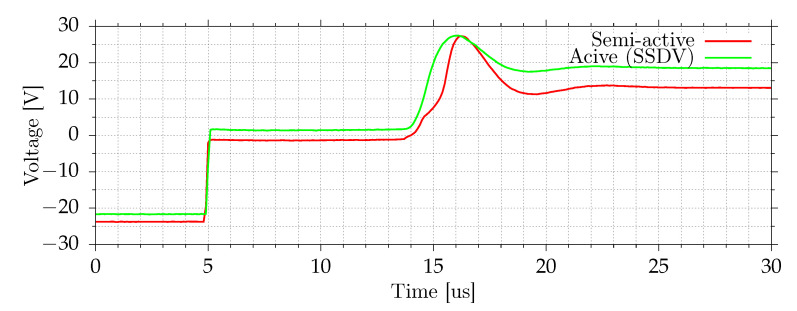
Voltage on a switch during negative to positive voltage transition.

Figure 13 shows the plate velocity measured by the vibrometer and the voltage on a switch. In the case of semi-active and active systems, this voltage is equal to the voltage on MFC elements and the inductor. The passive system has an inductor between the switching circuit and the ground, and in that case, the presented voltage is equal to the voltage on MFC elements only. When the current does not flow in the circuit (switch is in high-impedance state), the voltage on induction is equal to zero. The disturbance frequency is equal to the open-circuit resonant frequency. All tested shunt systems reduce vibrations in this case. Figure 14 and Figure 15 show the voltage changes during switching. Except for the transition, where voltage boost is used, the switching efficiency in this case is in the 55–65% range. For lower amplitudes, the switching efficiency drops. During the switch, the voltage is equal to about 0.5–0.7 V, mostly due to series diode. With voltage boost, the efficiency increases to 85%. For lower amplitudes, the efficiency can be even higher because boost voltage, 3.3 V, would be higher compared to voltage on MFCs.

### 3.3. Disturbance Level Dependence

All tested shunt systems behave nonlinearly. The switching is inherently nonlinear, but there are also other sources of nonlinearity, which affect performance at some levels. The switches with diode protection against reverse currents have static nonlinearity. For very small voltages on MFC, the voltage may be lower than diode’s forward voltage, and the switching current will be close to zero. In the passive circuit, there is also a voltage drop on the Darlington pair and on the maxima detector. In semi-active and active circuits as well, the digital maxima detector is nonlinear. At higher vibration levels, the mechanic structure itself behaves nonlinearly. In this research, however, the excitation levels were chosen to avoid speaker and mechanic nonlinearities and do not correspond to any real-world application.

**Figure 16:**
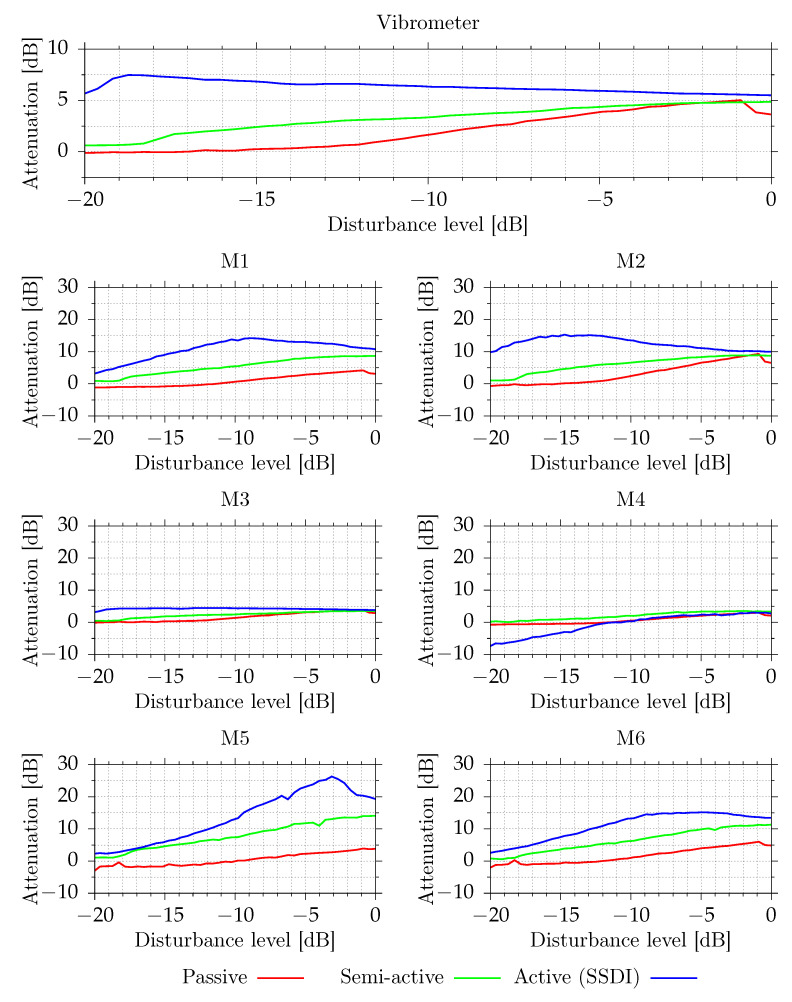
Measured vibroacoustical signal powers for different excitation level at open circuit resonance.

To test dependence on disturbance level, a tonal disturbance with a frequency equal to open circuit resonance was used. The amplitude of disturbance was changed to the −20 dB to 0 dB range. The results are presented in Figure 16.

The passive system achieves the best performance when the disturbance level is equal to ca. −1 dB. Below −14 dB, the system is not effective due to internal voltage drops. Above −1 dB, the performance degrades. Without C2 and C3 capacitors, the performance starts to degrade by −7 dB and only 3.0 dB vibration reduction is obtained. Even higher capacitance of C2 and C3 capacitors may be required to avoid the degradation. The value of C2 and C3 capacitors was chosen based on simulations [53]. In this paper, the improvement has been experimentally confirmed.

The semi-active system’s performance increases with increasing amplitude, as expected. The active system achieves its best vibration reduction performance at quite low disturbance level, around −19 dB. At higher disturbance levels, the performance slowly drops to the performance of a semi-active circuit. For very large disturbance levels, the voltages of MFCs are very high, and a 3.3 V voltage boost has only small positive effect.

### 3.4. Performance for a Wide Range of Frequencies

**Figure 17:**
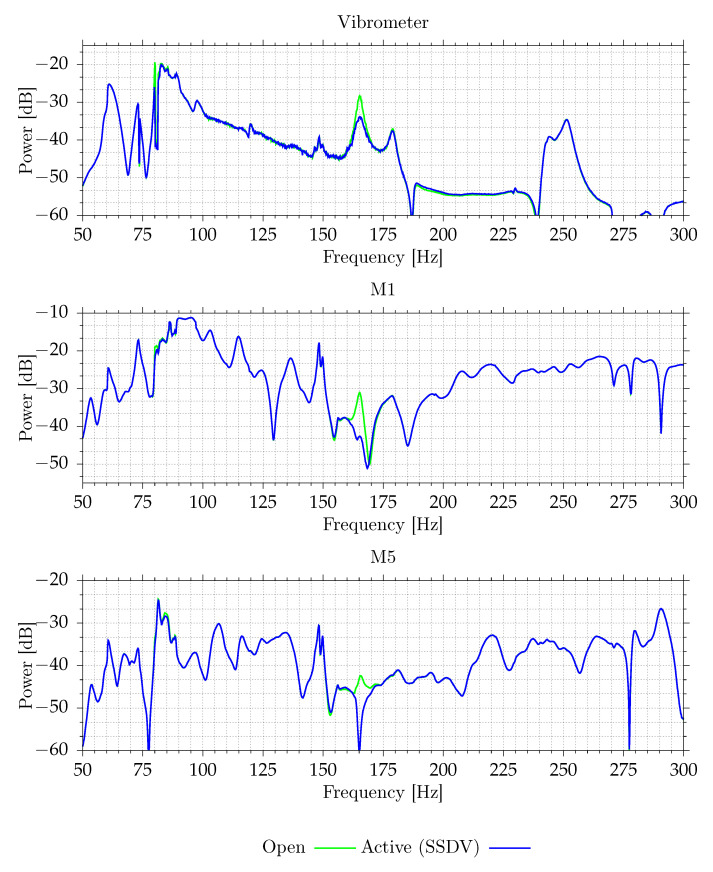
Measured vibroacoustical signal powers of selected signals for different excitation frequencies.

Figure 17 shows the performance of an SSDV circuit for a wider range of frequencies. Due to the series connection of MFC patches, the circuit is only effective for frequencies close to the natural frequency of the selected vibration mode. This mode has the highest vibration magnitude in the 100 Hz to 300 Hz range. For this mode, both vibration and SPL reduction are observed. However, acoustically, there are many frequency bands with higher noise transmission. Some of them are related to other noise propagation paths. For instance, the peak at 145–150 Hz is related to transmission though other walls, which are made of 3 mm aluminum plates. The FEM model predicts that the first resonant frequency should be equal to 148.6 Hz.

## 4. Discussion

The presented results show that all proposed circuits provide measurable vibration and SPL reduction for the selected mode. The active circuit, as expected, provides the best performance. Up to 5.5 dB vibration reduction was observed, when compared to the system with installed MFC actuators in the open state at the assumed maximal excitation level. For lower excitation levels, due to added additional energy, up to 7.5 dB vibration reduction was observed, as well as reduction of SPL. The SPL reduction levels, however, are very dependent on the specific location. At microphones close to the plate, up to 10 dB SPL reduction was observed for high excitation levels, and up to 15 dB for lower levels, which is a very good result as compared to classical, much more expensive fully active methods.

The semi-active system provides slightly lower vibration reduction at high excitation levels when compared to the active system. Up to 4.9 dB vibration reduction was achieved. For lower excitation levels, the difference increases. For low excitation levels, where the active circuit provides the best performance, vibration reduction is very low, smaller than 1 dB. This performance reduction is caused by voltage drops on the switch. The SPL reduction is close to active circuit for high excitation levels but decreases for lower levels.

The passive circuit behaves similarly to the semi-active system, but due to higher voltage drops in the circuit, it requires much higher excitation levels to operate. For high vibration levels, this system is very attractive due to a fully passive operation. This system does not require an external energy source. The energy required for switching control comes from vibrations.

The active circuit that implements the SSDV approach requires only small changes compared to the semi-active SSDI circuits. No additional elements are needed. Therefore, the semi-active to active upgrade is virtually free. For higher excitation levels, the used 3.3 V boost voltage is too small. The performance could be improved by increasing this voltage and also by providing a boost for the other voltage transition, from positive voltage to negative. This change requires additional supply voltage, for instance −3.3 V. In the proposed circuit, the boost voltage is limited by gate-to-source maximal voltage. The gate-to-source voltage in the used MOSFETs must be less or equal to 20 V. Because, in the proposed circuits, +15 V and −15 V voltages are used to drive the gate the maximal boost, voltage is equal to 5 V. For higher boost voltages, lower voltage must be used to gate control.

## 5. Conclusions

This paper presents research on three types of vibration damping circuits: passive SSDI, semi-passive SSDI and active SSDV. The research constitutes an extension of previously developed simulations and conducted experiments dedicated to structural vibration reduction.

In their previous experiments [18], the authors considered vibration reduction only. In this research, acoustic performance of each shunt system was also investigated through the SPL measurement with the use of microphones. Moreover, the authors improved previously used passive SSDI circuits and introduced new, semi-active version of SSDI and active SSDV circuits.

The research indicates that the active system provides the highest reduction of the velocity of structure movement at the point of measurement around the selected resonant frequency, equal to 5.5 dB in comparison to the open circuit. The level of SPL reduction is dependent on the locations of microphones and reached 15 dB for low excitation levels for the microphones close to the plate. The active system has in general the best performance in relation to the disturbance level. However, for higher levels of disturbance, performance of semi-active and active systems becomes similar.

The results are promising and may be a good starting point for further development of controllers to be employed in semi-active and active systems for noise and vibration reduction. The presented approach may be used in the casings enclosing noise-generating devices, not only to reduce structural vibration, but, as was proven, the noise may be significantly reduced around the casing as well.

## Figures and Tables

**Table 1:** The coordinates of the microphones and vibrometer. All dimensions are given in meters.

Sensor	M1	M2	M3	M4	M5	M6	V
x	0.67	0.32	0.30	0.03	1.31	0.43	0.30
y	0.81	1.03	0.97	0.81	3.16	2.26	2.86
z	0.26	0.23	0.64	0.22	1.49	1.36	0.46

**Table 2:** Parameters of the plate and the MFC elements.

Property	Plate	MFC
Dimensions [mm]	420 × 420 × 0.98	85 × 14 × 0.3
Mass [g]	463	2.0
Density [kg/m${}^{3}$]	2680	5440
Young’s modulus [GPa]	70.5	30.3/15.8
Poisson’s ratio	0.33	0.31

**Table 3:** Measured signal powers and reduction compared to the open circuit, for the open circuit resonant frequency. For the passive circuit, for which open circuits performance is not available, the short circuit is used instead.

Sys.	Freq. [Hz]	V [dB]	M1 [dB]	M2 [dB]	M3 [dB]	M4 [dB]	M5 [dB]	M6 [dB]
Short	166.5	−27.8	−34.4	−39.9	−40.9	−32.2	−50.5	−48.6
Passive	166.5	−31.6	−36.6	−47.2	−43.8	−33.9	−50.2	−51.2
Reduction	166.5	+3.8	+2.2	+7.3	+2.9	+1.7	−0.2	+2.6
Open	166.6	−27.4	−34.4	−36.9	−37.0	−33.9	−43.4	−45.3
Short	166.6	−27.6	−35.1	−36.7	−37.2	−37.2	−43.8	−45.8
Semi-active	166.6	−32.1	−42.5	−45.6	−40.7	−36.2	−56.5	−56.5
Reduction	166.6	+4.7	+8.1	+8.8	+3.7	+2.3	+13.1	+11.2
Open	165.9	−27.7	−34.6	−36.9	−38.3	−35.0	−44.2	−45.4
Active	165.9	−33.2	−45.7	−47.0	−42.1	−37.8	−64.5	−59.0
Reduction	165.9	+5.5	+11.1	+10.1	+3.8	+2.8	+20.3	+13.7

## Abbreviations

The following abbreviations are used in this manuscript:

ANC	Active Noise Control
ANVC	Active Noise-Vibration Control
ASAC	Active Structural Acoustic Control
PCB	Printed Circuit Board
SSD	Synchronized Switch Damping
SSDS	Synchronized Switch Damping on Short circuit
SSDC	Synchronized Switch Damping on Capacitor
SSDD	Synchronized Switch Damping with Diode
SSHI	Synchronized Switch Harvesting on Inductor
SSDI	Synchronized Switch Damping on Inductor
SSDV	Synchronized Switch Damping on Voltage source
SSSA	Synchronized Switch Shunt Architecture
SSDNC	Synchronized Switch Damping on Negative Capacitance
BJT	Bipolar Junction Transistor
MFC	Macro Fiber Composite
PSD	Piezoelectric Shunt Damping
AVC	Active Vibration Control
DOF	Degrees of Freedom
MOSFET	Metal-Oxide Semiconductor Field Effect Transistor
ADC	Analog to Digital Converter
SPL	Sound Pressure Level
